# Direct Computation of 3-D Stress Intensity Factors of Straight and Curved Planar Cracks with the P-Version Finite Element Method and Contour Integral Method

**DOI:** 10.3390/ma14143949

**Published:** 2021-07-15

**Authors:** Jianming Zhang, Rui Xu, Yong He, Wensheng Yang

**Affiliations:** 1 Department of Engineering Mechanics, Kunming University of Science and Technology, Kunming 650500, China; 20192110010@stu.kust.edu.cn (R.X.); 20192110044@stu.kust.edu.cn (W.Y.); 2 Yunnan Institute of Water and Hydropower Engineering Investigation, Design and Research, Kunming 650032, China; heyong2@ynwdi.com

**Keywords:** 3-D stress intensity factors, T-stress, p-version finite element method, contour integral method, numerical simulation

## Abstract

This paper presents direct computations of 3-D fracture parameters including stress intensity factors (SIFs) and T-stress for straight and curved planar cracks with the p-version finite element method (P-FEM) and contour integral method (CIM). No excessive singular element or enrichment function is required for the computation. To demonstrate the accuracy and efficiency of the proposed approaches, several benchmark numerical models of 3-D planar straight and curved cracks with single and mixed-mode fractures are considered and analyzed: a through thickness edge straight crack in a homogeneous material, a through thickness inclined straight crack, a penny-shaped crack embedded in a cube and a central ellipse shaped crack in a homogeneous cube. Numerical results are analyzed and compared with analytical solutions and those reported by the extended finite element method (XFEM) and the scaled boundary finite element method (SBFEM) in the available literature. Numerical experiments show the accuracy, robustness and effectiveness of the present method.

## 1. Introduction

Inherent flaws or cracks in complex engineering materials and structures are unavoidable. The existence of cracks will affect the safety and durability of structures. The analysis of 3-D crack behavior is of great significance in practical engineering. The stress intensity factors are important fracture parameters in linear elastic fracture analysis, which are often applied to characterize the displacements and stresses fields near the crack tip and further to predict crack propagations. In an analysis of three-dimensional fracture problems, the SIFs *K*_I_, *K*_II_ and *K*_III_ accurately describe the stress state at the crack tip. The accurate computation of 3-D fracture parameters (including SIFs and T-stress) for straight or curved planar cracks with single and mixed-mode loading is very important and has always been a challenging issue in linear elastic fracture analysis.

Several numerical methods have been proposed in the literature to extract 3-D stress intensity factors, including the meshless method (MLM) [1,2,3], the enriched finite element method (EFEM) [4,5,6,7], the extended/generalized finite element method (XFEM)/(GFEM) [8,9,10,11,12,13,14] and the scaled boundary finite element method (SBFEM) [15].

Classical problems in 3-D fracture mechanics include: a through thickness edge straight crack in a homogeneous specimen, a through thickness inclined crack, a penny-shaped crack in a homogeneous rectangular plate and an ellipse shaped crack embedded in a homogeneous large cube. A lot of techniques are proposed to analyze the aforementioned fracture problems. Numerical results of the SIFs for a through thickness edge straight crack in a homogeneous specimen have been reported by using different numerical methods in [1,13,14]. Wang et al. [13] evaluated the three-dimensional SIFs of an edge parallel crack specimen in a rectangular block using the adaptive multiscale XFEM and interaction integral method. Wang et al. [14] computed the SIFs of an edge straight crack in a cube and a central ellipse shaped crack in a large cube using local mesh refinement in combination with the XFEM and greatly improved the accuracy of SIFs computed by the classical extended finite element method. Wang et al. [14] also studied a mixed-mode edge inclined straight crack in a plate and considered the effects of various crack lengths for the SIFs. Saputra et al. [15] computed the SIFs and T-stress of a penny-shaped crack in a homogeneous cube using the scaled boundary finite element method (SBFEM).

Bhat et al. [16] analyzed the effect of specimen parameters on mixed-mode I/II SIFs for additive manufactured slant edge crack plate. The mixed-mode I/II stress intensity factors were studied for an inclined crack in a specimen. The authors observed that the specimen parameters, the crack length ratio, crack angle and layer-built angle significantly affect the stress intensity factors of slant edge crack plate. Khosravani et al. [17] experimentally investigated fracture characteristics and load-carrying capacity of 3-D-printed components (polycarbonate and nylon filaments) produced using fused deposition modeling, and their numerical simulation results are in very good agreement with reported experimental results. The above studies are important for the domain of manufacturing and engineering. We aim to generalize the present method to analyze similar industrial applications problems in our next manuscript.

The objective of this paper is to propose an effective numerical approach to accurately estimate the stress intensity factors (SIFs) and T-stress for 3-D planar straight and curved cracks. Especially, for the accurate computation of 3-D fracture parameters, which is a challenging task in 3-D cracked structures, the numerical approach is able to improve the accuracy of fracture parameters compared to other numerical methods. In this paper, the fracture parameters, including the stress intensity factors (SIFs) and T-stress for 3-D planar straight and curved cracks with single and mixed-mode of several benchmark problems, were computed directly using P-FEM in combination with CIM. The validity and accuracy of the present approaches are illustrated by numerical models, and numerical results were analyzed and compared with analytical solutions and published results obtained by the adaptive multiscale extended finite element method (XFEM), local mesh refinement XFEM (Lm-XFEM) and scaled boundary finite element method (SBFEM) [13,14,15]. Computation examples show that the SIFs computed by the present method have faster convergence speed, smaller relative error and better stability.

## 2. P-Version Finite Element Method and Contour Integral Method in Three Dimensions

### 2.1. P-Version Finite Element Method in Three Dimensions

Approximation theory of the three-dimensional p-version of the finite element method has been established [18,19,20]. The p-version of the finite element method has turned out to be an efficient discretization technique for many engineering problems. The p-version FEM has an exponential rate of convergence in energy norm with a proper mesh design. Generally, the p-version FEM is superior to the traditional h-version FEM. The application of the p-version FEM in three dimensions, including in three -dimensional linear elastic fracture analysis, can be found in [21,22,23,24,25,26,27].

### 2.2. 3-D Hierarchical Shape Functions for Standard Tetrahedral Elements

**Figure 1:**
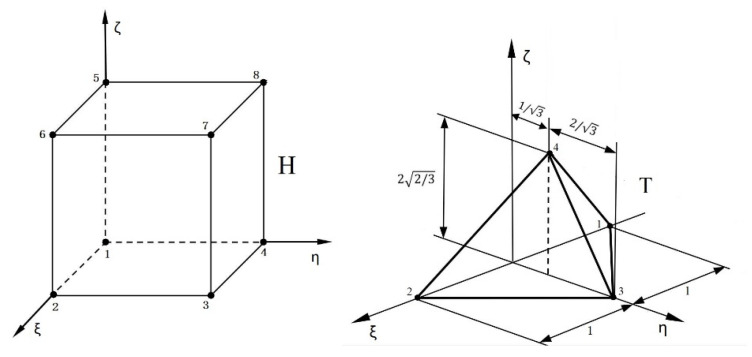
Standard hexahedral element H and standard tetrahedral element T.

There are several element types for three-dimensional problems: the hexahedral, tetrahedral, pentahedral (wedge), and pyramid elements. The most common elements are the hexahedral and tetrahedral elements (shown in Figure 1).

There are four types of hierarchical shape functions of the three-dimensional p-version FEM on standard tetrahedral element T: (1) nodal modes shape functions, (2) edge modes shape functions, (3) face modes shape functions and (4) internal modes shape functions. Details about these shape functions defined on a standard tetrahedral element can be found in [28]. In this paper, we use tetrahedral elements to compute fracture parameters, and we find the results obtained from tetrahedral elements are better than those from hexahedral elements.

### 2.3. Contour Integral Method in Three Dimensions

Szabó et al. [29] first reported the contour integral method as a super-convergent technique to calculate stress intensity factors. The contour integral method can be utilized to calculate modes I, II and III SIFs. Garzon et al. [9] applied CIM in combination with the generalized finite element method to extract the SIFs of 3-D crack. The three-dimensional formulae used in [9] are as follows:

(1) $$K_{I}=\sum_{i=1}^{2}\left(\int_{\Gamma_{2}}T_{i}^{\left(u\right)}v_{i}^{-I}d\Gamma -\int_{\Gamma_{2}}T_{i}^{\left(v^{-I}\right)}u_{i}d\Gamma +\int_{\Gamma_{3}}p_{i}^{3}v_{i}^{-I}d\Gamma +\int_{\Gamma_{4}}p_{i}^{4}v_{i}^{-I}d\Gamma \right)$$

(2) $$K_{\mathrm{II}}=\sum_{i=1}^{2}\left(\int_{\Gamma_{2}}T_{i}^{\left(u\right)}v_{i}^{-II}d\Gamma -\int_{\Gamma_{2}}T_{i}^{\left(v^{-II}\right)}u_{i}d\Gamma +\int_{\Gamma_{3}}p_{i}^{3}v_{i}^{-II}d\Gamma +\int_{\Gamma_{4}}p_{i}^{4}v_{i}^{-II}d\Gamma \right)$$

(3) $$K_{\mathrm{III}}=\int_{\Gamma_{2}}T_{3}^{\left(u\right)}v_{3}^{-III}d\Gamma -\int_{\Gamma_{2}}T_{3}^{\left(v^{-III}\right)}u_{3}d\Gamma +\int_{\Gamma_{3}}p_{3}^{3}v_{3}^{-III}d\Gamma +\int_{\Gamma_{4}}p_{3}^{4}v_{3}^{-III}d\Gamma$$

where $v_{i}^{-I}$, $v_{i}^{-II}$ and $v_{i}^{-III}$ represent separately the extraction functions of modes I, II and modes III SIFs, $T_{i}^{\left(v^{-I}\right)}$, $T_{i}^{\left(v^{-II}\right)}$ and $T_{i}^{\left(v^{-III}\right)}$ are separately the traction vectors computed from $v_{i}^{-I}$, $v_{i}^{-II}$ and $v_{i}^{-III}$. A detailed analysis of the 3-D CIM can be found in [9]. The stress intensity factors *K*_I_, *K*_II_ and *K*_III_ in (1), (2) and (3) are exact solutions, but *u* and the corresponding traction vector *T^(**u**)^* are unknown. Any numerical method can be used to approximate the exact solutions *u*. In this study, numerical solutions calculated by the three-dimensional p-version finite element method are used to replace the exact solutions *u*, the corresponding traction vectors calculated are used to replace the exact traction *T^(**u**)^* and the approximate solutions of *K*_I_, *K*_II_ and *K*_III_ are finally obtained.

## 3. Numerical Examples and Discussion

In order to illustrate the effectiveness and the accuracy of the present approaches, the SIFs and T-stress of three-dimensional planar straight and curved cracks in 3-D objects were computed for the following four crack configurations: (1) a through thickness edge straight crack in a homogeneous material; (2) a mixed-mode edge inclined straight crack in a rectangular block; (3) a penny-shaped crack embedded in a cube; and (4) an ellipse shaped crack embedded in a cube.

Based on the 3-D p-version FEM and CIM, numerical models are presented in two kinds of problems: planar straight cracks and planar curved cracks in 3-D objects with single and mixed-mode fractures. The first two models are used to investigate single and mixed-mode fractures in 3-D objects by studying an edge straight crack and an edge inclined straight crack and to verify the validity and accuracy of the present method by comparing numerical results with analytical solutions [30] and those obtained by the adaptive multiscale extended finite element method [13], local mesh refinement extended finite element method [14] and scaled boundary finite element method [15]. In the last two examples, the 3-D p-version finite element method and contour integral method are applied to investigate planar curved cracks in 3-D by studying a central penny-shaped crack and a central elliptical crack and to demonstrate the accuracy and effectiveness by comparing numerical results with reference solutions [31,32] and those obtained by the scaled boundary finite element method [15] and the local mesh refinement extended finite element method [14]. All examples can be compared with those published in the literature to evaluate the accuracy of the present method; these examples show the simplicity in computation of the SIFs without the need to use excessive singular elements or enrichment functions near the crack front.

### 3.1. An Edge Straight Crack

In this subsection, an example of an edge straight crack in a rectangular block is presented to demonstrate the efficiency and accuracy of the present approach. The same example has been investigated by Wang et al. [13] and Saputra et al. [15]. The SIFs *K*_I_ are computed by the p-version FEM in combination with CIM and compared with the analytical solutions and results reported by the adaptive multiscale extended finite element method and the scaled boundary finite element method. This example is considered under the plane strain condition.

#### SIFs of an Edge Straight Crack in a Rectangular Block

**Figure 2:**
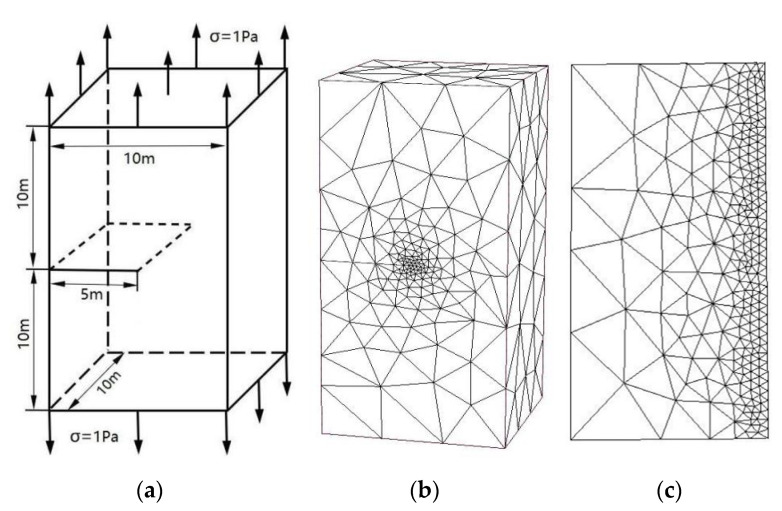
(**a**) An edge straight crack specimen; (**b**) present mesh discretizing of an edge straight crack (total node numbers: 26,131, total element numbers: 18,447); (**c**) details of mesh discretization near crack front.

Consider a rectangular specimen of size 10 m × 10 m × 20 m which contains an edge straight crack as shown in Figure 2a. The crack length is 5 m; the top surface and bottom surface of the specimen are subjected to a unidirectional uniform traction of σ=1 Pa. The material parameters are considered for E=206 GPa and ν=0.3 (Young’s modulus and Poisson’s ratio). The analytical solution of the SIFs *K*_I_ for the edge straight crack problem is given by [30] under the plane strain condition and $\frac{d}{l}\leq 0.6$:

$$K_{I}=\sigma \sqrt{\pi d}F(\frac{d}{l})$$

where d is the crack length, l is the length of the rectangular block along the crack direction and $F=1.12-0.231\frac{d}{l}+10.55{\left(\frac{d}{l}\right)}^{2}-21.72{\left(\frac{d}{l}\right)}^{3}+30.39{\left(\frac{d}{l}\right)}^{4}$.

Figure 2b,c shows the P-FEM mesh of the edge straight crack problem and the present local mesh refinements in the crack front.

**Figure 3:**
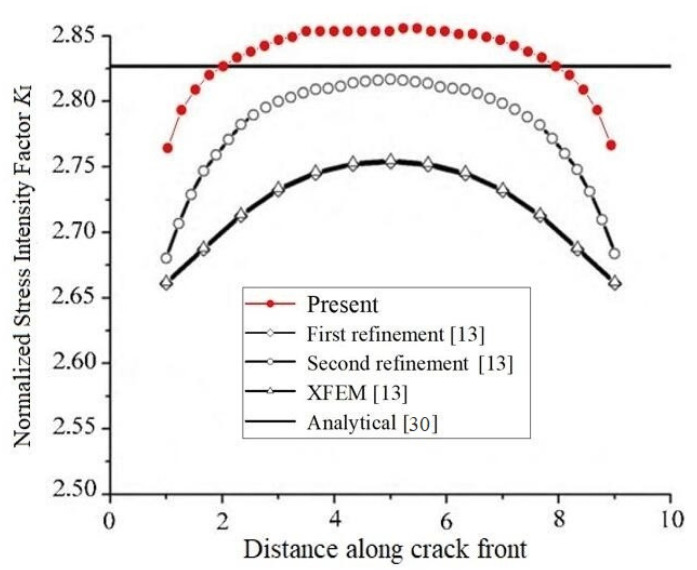
Comparison of the normalized SIFs *K*_I_ at different distances along the crack front for an edge straight crack in a plate among the present method, adaptive multiscale extended finite element method, convectional XFEM and analytical solution.

The SIFs *K*_I_ computed by the present approach and the analytical solutions are listed in Table 1. A comparison with the results obtained by the adaptive multiscale extended finite element method in [13] are shown in Figure 3.

In this example, the normalized analytical SIFs *K*_I_ = 2.826. Obviously, from Figure 3, the present SIFs *K*_I_ are closer to the analytical solutions at most points of the crack front.

**Figure 4:**
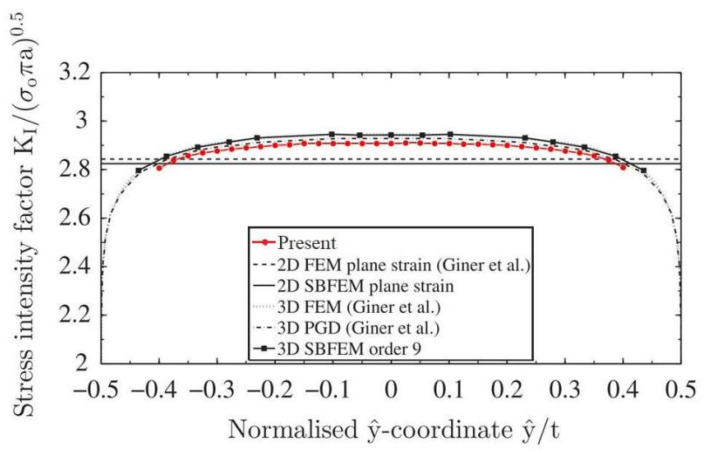
Comparison of the normalized SIFs *K*_I_ at different distances along the crack front for an edge straight crack in a plate among the present method, SBFEM, 3D FEM, 3D PGD and the analytical solution.

A similar model has been considered by Saputra et al. [15]. At the same conditions, namely, when elastic material properties are the same as Young’s modulus E and Poisson’s ratio ν=0.3, when uniform uniaxial tension is subjected at the top and bottom surfaces of the specimen and when the other boundaries are traction free, the SIFs and T-stress are to be calculated by the present method. The meshes for the present method are shown in Figure 2b,c. Comparisons with the results obtained by the scaled boundary finite element method in [15] are shown in Figure 4.

In the computation of the example, the number of discretizing meshes is 18,447, and the degrees of hierarchical shape functions are p=1,⋯,8**.** The results of *K*_I_ along the crack front are presented in Figure 4. The present results are in very good agreement with the results of the SBFEM obtained with element order 9 and are closer to the analytical solution.

### 3.2. An Edge Inclined Straight Crack

**Figure 5:**
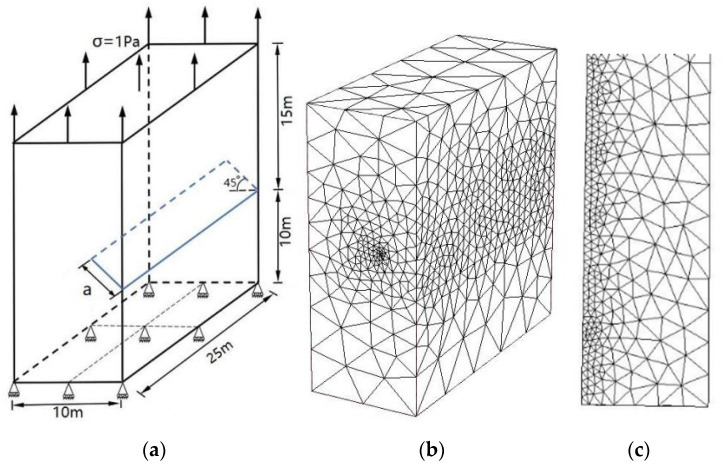
(**a**) An edge inclined straight crack in a rectangular specimen; (**b**) present mesh discretizing of an edge inclined straight crack (total node number is 47,404, total element number is 33,292); (**c**) details of mesh discretization near crack front.

Figure 5a shows an example of an edge inclined straight crack in a rectangular specimen subjected to uniform tension. The example aims to investigate the effect of different crack lengths for SIFs of the mixed-mode in 3-D planar straight cracks. The dimensions of the specimen are 10 m × 25 m × 25 m, and the angle of the edge inclined crack is $45^{\circ }$. The material parameters are used for E=206 GPa and ν=0.3 (Young’s modulus and Poisson’s ratio). The top surface of the plate is applied to a uniform traction of σ=1 Pa; the bottom surface is constrained in all directions. The length of the crack is 25 m.

Figure 5b,c shows the P-FEM mesh of the edge inclined straight crack and the present local mesh refinements in the crack front.

**Figure 6:**
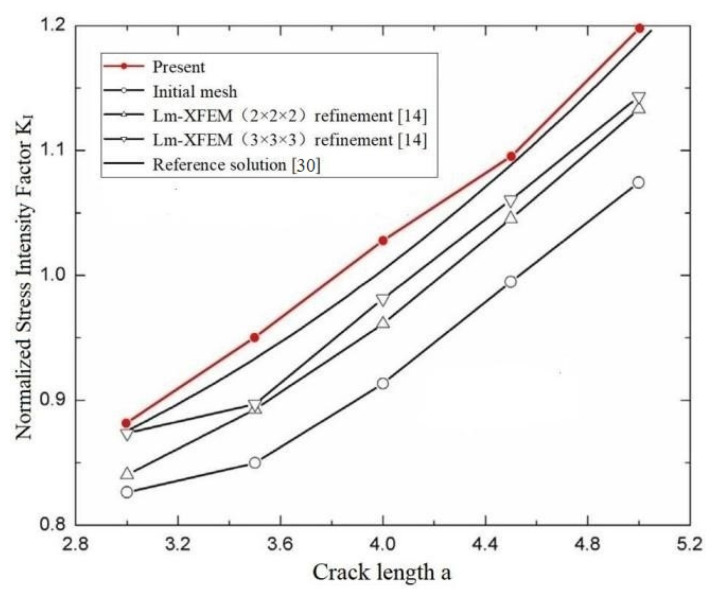
Comparison of the normalized SIFs *K*_I_ of an edge inclined straight crack in a cube among the present method, conventional XFEM, Lm-XFEM and the reference solution.

**Figure 7:**
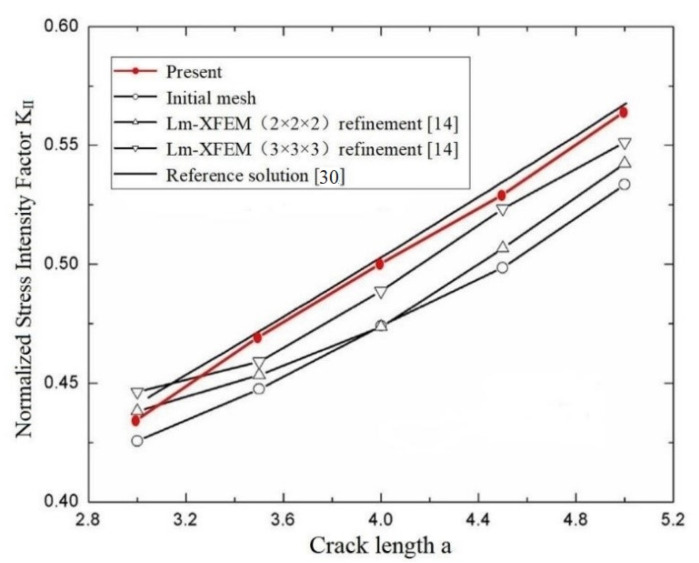
Comparison of the normalized SIFs *K*_II_ of an edge inclined straight crack in a cube among the present method, convectional XFEM, Lm-XFEM and the reference solution.

Normalized SIFs *K*_I_ and *K*_II_ are computed for different crack lengths (a varies from 3 to 5) using the present method and are listed in Table 2 and Table 3. The reference solutions are given by the *Stress Intensity Factors Handbook* [30]. Comparisons with the reference solution and results of the normalized SIFs *K*_I_ and *K*_II_ in [14] are shown in Figure 6 and Figure 7.

In the computation of the example, the number of discretizing meshes is 33,292, and the degrees of hierarchical shape functions are *p* from 1 to 8. It is easy to see from Figure 6 and Figure 7 that the present results of normalized SIFs *K*_I_ and *K*_II_ are closer the reference solutions.

### 3.3. A Penny-Shaped Crack

**Figure 8:**
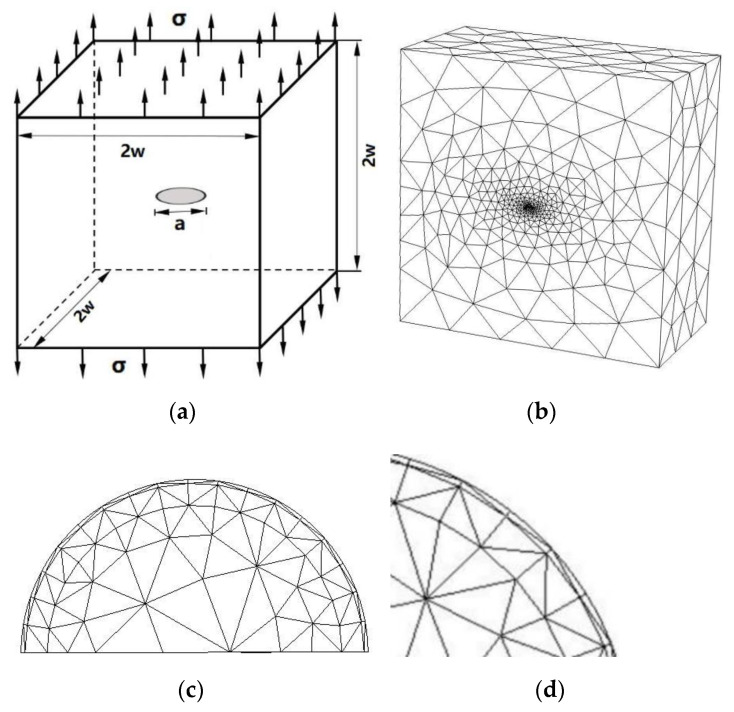
(**a**) A central penny-shaped crack in a homogeneous material; (**b**) present discretization of meshes (one-half of the penny-shaped crack; total node number is 46,054; total element number is 33,478); (**c**) details of mesh discretization near crack front (one-half of the penny-shaped crack); (**d**) local details of discretization.

Accurate computation of 3-D fracture parameters (such as SIFs and T-stress) for curved cracks is a challenging problem in linear elastic fracture analysis. Many efforts have been made to improve the accuracy of 3-D fracture parameters in the literature in the past two decades. A central penny-shaped crack with radius a in a homogeneous material is investigated as shown in Figure 8a. The dimensions of the model are a/w=0.08, w=80. The material parameters are used for *E* and ν=0.3 (Young’s modulus and Poisson’s ratio).

A uniaxial uniform tension σ=1 Pa is subjected to the top and bottom surfaces of the cube. In this example, the p-version FEM and CIM are applied to extract 3-D SIFs and T-stress for the planar curved cracks in three dimensions. Figure 8b–d shows the present p-version FEM mesh of the penny-shaped crack and the local mesh discretization in the crack front. The analytical solutions of the stress intensity factor *K*_I_ and the T-stress *T* in the penny-shaped crack in an unbounded domain are given in [31,32]:

(4) $$K_{I}=\frac{2}{\pi }\sigma \sqrt{\pi a\;},T=-\frac{1+2\nu }{2}\sigma .$$

**Figure 9:**
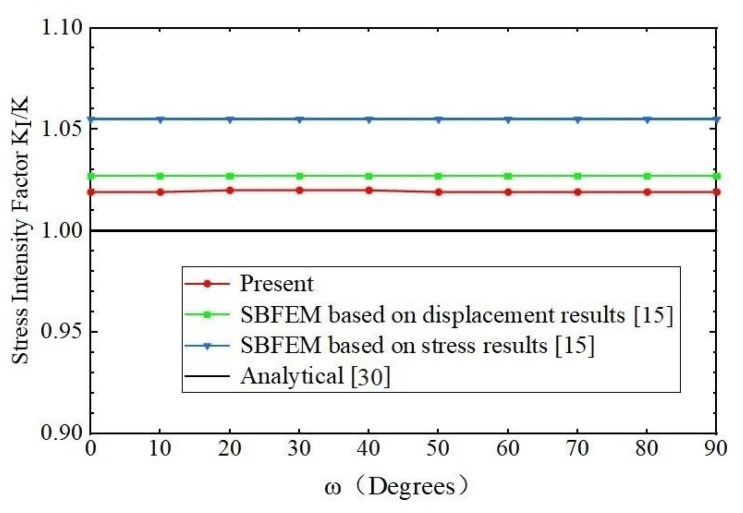
Comparison of the SIFs *K*_I_ of a penny-shaped crack in a homogeneous material among the present method, SBFEM based on displacement, SBFEM based on stress and analytical solution.

**Figure 10:**
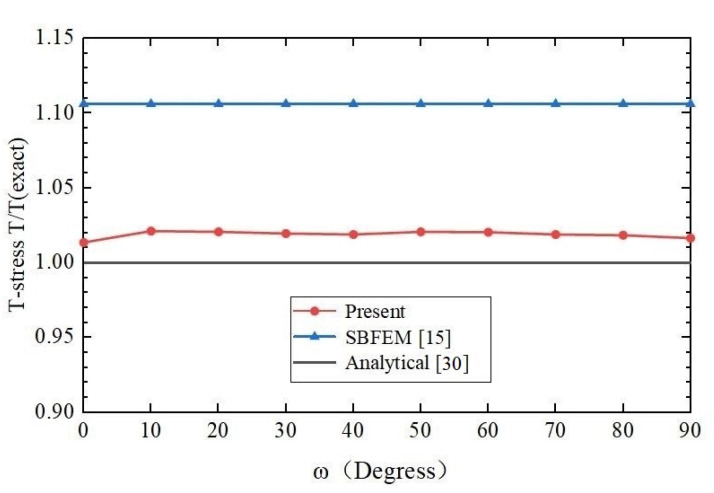
Comparison of the T-stress *T* of a penny-shaped crack in a homogeneous material among the present method, SBFEM and analytical solution.

The analytical solutions (4) are used as the reference solutions in the model. The SIFs *K*_I_ and T-stress *T* of the penny-shaped crack calculated by the present method, the scaled boundary finite element method [15] and analytical solutions are compared in Table 4 and Table 5 separately and shown in Figure 9 and Figure 10.

In Table 4 and Table 5, as the computation values of SIFs *K*_I_ and T-stress *T* are not given in [15], only the differences of the computed values *K*_I_ and *T* to the exact values are listed from [15]. In the computation of the example, the number of discretizing meshes is 33,478, and the degrees of hierarchical shape functions are *p* from 1 to 8. It is easy to see from Table 4 and Table 5 that the relative errors of the SIFs *K*_I_ computed by the present method are less than and equal to 2%, and the relative errors of the T-stress *T* computed by the present method are less than 2.15%. All of them are less than the results of the SBFEM.

Table 6 shows that the errors of energy norm and relative errors of SIF *K*_I_ for interpolation polynomial order *p* from 1 to 8. It is easy to see that the error of energy norm and relative error of SIF *K*_I_ gradually decrease with the increasing of the polynomial degree p, and the errors gradually stabilize. Other examples are similar to this one; for simplicity, we list only Table 6 for reference.

### 3.4. A Central Ellipse Shaped Crack

#### SIFs of a Central Ellipse Shaped Crack in a Large Cube

**Figure 11:**
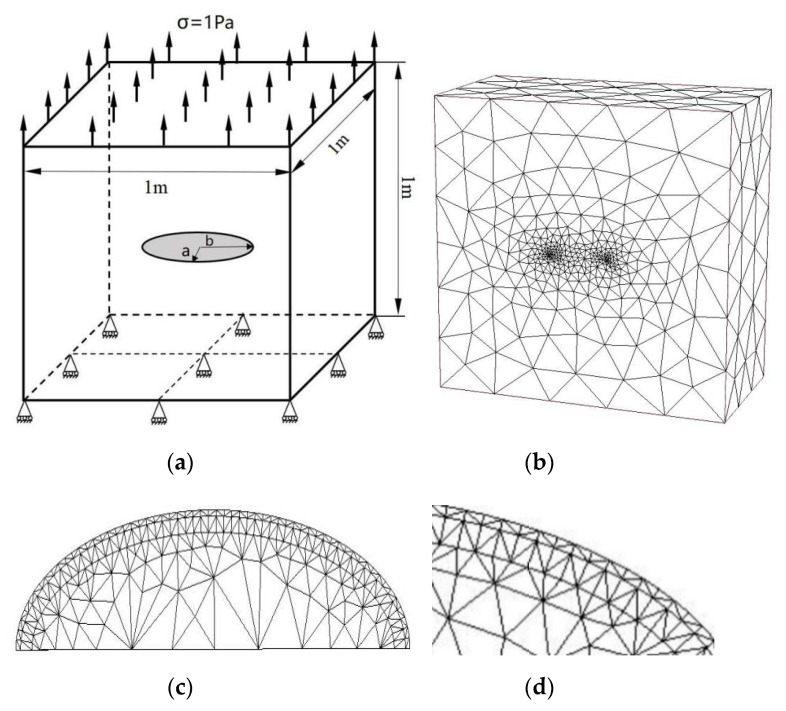
(**a**) A central ellipse shaped crack in a homogeneous large cube; (**b**) present mesh discretizing one-half of the central ellipse (total node number is 49,406; total element number is 36,247); (**c**) details of mesh discretization in the central ellipse shaped crack front (one-half of the central ellipse shaped crack front); (**d**) local details of discretization.

A central ellipse shaped crack in a cube subjected to a uniaxial uniform traction on the top surface and the bottom surface constrained in all directions as shown in Figure 11a are considered. The dimensions of the cube are 1 m ×1 m × 1 m, and the semi-major axis and semi-minor axis of the ellipse shaped crack are b = 0.1 m and a = 0.05 m. The material parameters are used for E=206 GPa and ν=0.3 (Young’s modulus and Poisson’s ratio).

Figure 11b–d shows the present mesh discretizing of the central ellipse shaped crack problem and the local mesh refinements near the crack front.

The exact SIFs for a central ellipse shaped planar crack in an infinite domain is listed in [32]:

(5) $$K_{I}=\sigma \sqrt{\pi a}\frac{{\left\{\sin^{2}\theta +\frac{b^{2}}{a^{2}}\cos^{2}\theta \right\}}^{1/4}}{E(k)}$$

where θ is the elliptic angle and E(k) is the second kind of elliptic integral,

(6) $$E(k)=\int_{0}^{\frac{\pi }{2}}\sqrt{1-k^{2}\sin^{2}\theta }d\theta ,\;k^{2}=\frac{b^{2}-a^{2}}{b^{2}}.$$

**Figure 12:**
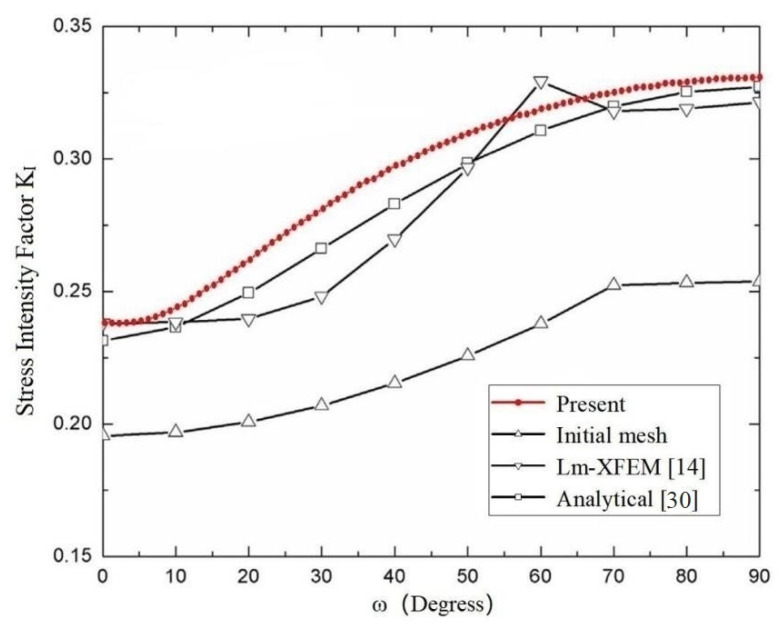
Comparison of the SIFs *K*_I_ of a central ellipse shaped crack in a homogeneous cube among the present method, conventional XFEM, Lm-XFEM and analytical solution for a=0.05 m, b=0.1 m. (a/b=0.5).

The model has been analyzed by Wang et al. [14] using the local mesh refinement extended finite element method, and excellent results have been obtained in [14]. The SIFs *K*_I_ computed by the present method and analytical solutions are given in Table 7, and a comparison with the analytical solutions and results of the SIFs *K*_I_ extracted by the local mesh refinement extended finite element method in [14] are shown in Figure 12.

Figure 12 shows clearly that the SIFs *K*_I_ extracted by the present method approximate the analytical solutions and are in good agreement with those obtained by the local mesh refinement extended finite element method. In the computation of the example, the number of discretizing meshes is 36,247, and the degrees of hierarchical shape functions are *p* from 1 to 8. It is easy to see that the relative errors of the SIFs *K*_I_ when 30≤ϖ≤60 are bigger than those when 0≤ϖ≤30 and 60≤ϖ≤90; the curve is more stable and there is no large jump.

## 4. Conclusions

The 3-D p-version FEM and contour integral method are applied in order to analyze 3-D fracture problems. The hierarchical high-order shape functions are used to approximate 3-D fracture parameters. No excessive singular element or enrichment function near the crack front is required for the computation. Numerical experiments show that the p-version FEM and CIM are efficient and accurate for the direct computation of stress intensity factors and T-stress in three dimensions. It is effective not only for straight planar cracks but also for curved planar cracks in the analysis of three-dimensional fracture problems. Numerical examples show that the fracture parameters determined by the present method are in very good agreement with the reported results in the literature and have higher precision and better numerical stability in most cases.

Although the present method is effective and straightforward for the computation of 3-D fracture parameters, it inevitably needs to remesh meshes near the crack front. Local mesh discretization or refinement is required in order to obtain results with higher accuracy. To overcome the drawback, it would be interesting if the present method could be combined with the extended finite element method to avoid remeshing.

## Figures and Tables

**Table 1:** Comparison of present SIFs *K*_I_ with analytical solutions.

Distance Along Crack Front (m)	Analytical *K*_I_ (Pa·m^1/2^)	Present *K*_I_ (Pa·m^1/2^)	Normalized *K*_I_ (Pa·m^1/2^)	Relative Error (%) [Present]
1	11.202	10.92	2.755	−2.50
2	11.202	11.18	2.821	−0.18
3	11.202	11.29	2.849	0.80
4	11.202	11.32	2.856	1.07
5	11.202	11.34	2.861	1.25
6	11.202	11.32	2.856	1.07
7	11.202	11.29	2.849	0.80
8	11.202	11.18	2.821	−0.18
9	11.202	10.93	2.758	−2.41

**Table 2:** SIFs *K*_I_ obtained by the present method for different crack lengths.

Distance Along Crack Front (m)	*K*_I_ (Pa·m^1/2^)				
	a = 3 m	a = 3.5 m	a = 4 m	a = 4.5 m	a = 5 m
0.9	2.703	3.153	3.603	4.168	4.730
1.8	2.707	3.148	3.648	4.124	4.730
2.7	2.704	3.167	3.675	4.162	4.757
3.6	-	-	3.606	4.036	4.753
4.5	-	-	-	-	4.769

**Table 3:** SIFs *K*_II_ obtained by the present method for different crack lengths.

Distance along Crack Front (m)	*K*_II_ (Pa·m^1/2^)				
	a = 3 m	a = 3.5 m	a = 4 m	a = 4.5 m	a = 5 m
0.9	1.320	1.557	1.794	1.959	2.188
1.8	1.334	1.612	1.746	1.945	2.237
2.7	1.321	1.548	1.826	1.990	2.262
3.6	-	-	1.768	1.941	2.179
4.5	-	-	-	-	2.198

**Table 4:** Analytical solutions and SIFs *K*_I_ obtained by different numerical methods.

Degrees	Analytical *K*_I_ (Pa·m^1/2^)	Present *K*_I_ (Pa·m^1/2^)	SBFEM [15] Stress Results Error (%)	SBFEM [15] Displacement Results Error (%)	Present Error (%)
0	1.1284	1.150	5.5	2.7	1.91
10	1.1284	1.150	5.5	2.7	1.91
20	1.1284	1.151	5.5	2.7	2.00
30	1.1284	1.151	5.5	2.7	2.00
40	1.1284	1.151	5.5	2.7	2.00
50	1.1284	1.150	5.5	2.7	1.91
60	1.1284	1.150	5.5	2.7	1.91
70	1.1284	1.150	5.5	2.7	1.91
80	1.1284	1.150	5.5	2.7	1.91
90	1.1284	1.150	5.5	2.7	1.91

**Table 5:** Analytical solutions and T-stress *T* obtained by different numerical methods.

Degrees	Analytical	Present	SBFEM [15] Results Error (%)	Present Error (%)
0	−0.8	−0.811	10.6	1.35
10	−0.8	−0.817	10.6	2.12
20	−0.8	−0.817	10.6	2.07
30	−0.8	−0.816	10.6	1.95
40	−0.8	−0.816	10.6	1.89
50	−0.8	−0.817	10.6	2.07
60	−0.8	−0.816	10.6	2.04
70	−0.8	−0.815	10.6	1.89
80	−0.8	−0.815	10.6	1.84
90	−0.8	−0.813	10.6	1.65

**Table 6:** DOF, error of energy norm and relative error of *K*_I_ by the present method.

*P*	DOF	Error of Energy Norm (%)	Analytical *K*_I_ (Pa·m^1/2^)	*K*_I_ (Pa·m^1/2^)	Relative Error (%) [Present]
1	18723	30.25	1.1284	0.810	−28.2
2	148194	17.28	1.1284	1.088	−3.58
3	497136	12.70	1.1284	1.141	1.12
4	1174290	10.16	1.1284	1.157	2.53
5	2288397	8.53	1.1284	1.154	2.27
6	3948198	7.39	1.1284	1.153	2.18
7	6262434	6.55	1.1284	1.153	2.18
8	9339846	5.90	1.1284	1.151	2.00

**Table 7:** Comparison of present SIFs *K*_I_ with analytical solutions.

Degrees	Analytical *K*_I_ (Pa·m^1/2^)	Present *K*_I_ (Pa·m^1/2^)	Present Error (%)
0	0.2314	0.2393	3.41
10	0.2365	0.2439	3.13
20	0.2495	0.2616	4.85
30	0.2661	0.2807	5.49
40	0.2831	0.2973	5.02
50	0.2983	0.3100	3.92
60	0.3107	0.3190	2.67
70	0.3198	0.3259	1.91
80	0.3254	0.3300	1.41
90	0.3273	0.3320	1.44

## Data Availability

Data is contained within the present article. Other data presented in this research are available in [13,14,15] and [30,31,32].
